# Zika virus and the fetal-maternal interface: deciphering the mechanisms of placental infection and implications for pregnancy outcomes

**DOI:** 10.1080/22221751.2025.2532681

**Published:** 2025-07-10

**Authors:** Sam Chak Sum Wong, Joshua Fung, Pak-Ting Hau, Yanjie Guo, Philip C. N. Chiu, Hong Wa Yung, Gilman Kit Hang Siu, Franklin Wang-Ngai Chow, Cheuk-Lun Lee

**Affiliations:** a Department of Health Technology and Informatics, The Hong Kong Polytechnic University, Hong Kong SAR, People’s Republic of China; b Department of Obstetrics and Gynaecology, The University of Hong Kong, Hong Kong SAR, People’s Republic of China; c Department of Obstetrics and Gynaecology, University of Cambridge, Cambridge, UK

**Keywords:** Zika virus (ZIKV), congenital Zika syndrome (CZS), microcephaly, placental infection, pregnancy, fetal-maternal interface, trophoblast

## Abstract

Zika virus (ZIKV) is an emerging flavivirus primarily transmitted by Aedes mosquitoes. It has gained significant attention due to its potential teratogenic effects during pregnancy, particularly the increased risk of severe congenital outcomes such as microcephaly and fetal mortality. ZIKV's ability to breach the fetal-maternal interface and compromise placental function distinguishes it from other arboviruses. This mini-review explores how ZIKV targets key placental cells, including cytotrophoblasts, extravillous trophoblasts, syncytiotrophoblasts, and Hofbauer cells, to establish infection in the placenta. We first explore the molecular mechanisms of ZIKV replication, focusing on viral entry, replication strategies, and evasion of host immunity in trophoblasts. We then discuss how ZIKV infection triggers cellular stress responses, such as oxidative stress, endoplasmic reticulum stress, and inflammatory pathways, along with metabolic reprogramming. These processes ultimately lead to placental insufficiency and adverse pregnancy outcomes. Drawing on recent findings from trophoblast organoids and in vivo models, we highlight novel therapeutic opportunities, including vasoactive intestinal peptide, endoplasmic reticulum stress modulators, and immunomodulatory interventions that may protect pregnancy. Finally, we propose future directions for mitigating ZIKV’s impact on maternal-fetal health, emphasizing the need for comprehensive research into viral pathogenesis, advanced model systems, and vector control programmes to prevent this devastating infection.

## Introduction

Zika virus (ZIKV) was first isolated in 1947 from a rhesus monkey used for yellow fever surveillance in Uganda's Zika Forest. By 1952, serological surveys had detected the virus in humans, revealing antibodies in 6% of tested individuals [1]. The virus remained relatively obscure for decades, with cases primarily reported in Africa and Asia. A turning point occurred in 2007 with an outbreak on Yap Island, affecting approximately 75% of the population [2]. The virus gained international attention during the 2015 outbreak in Brazil, which was linked to severe complications. In February 2016, the World Health Organization declared a Public Health Emergency, confirming the association between ZIKV infection during pregnancy and congenital malformations [3,4]. Although symptomatic cases of ZIKV infection are often mild, the teratogenic risk is profound. The virus can cross the placenta, causing congenital Zika syndrome (CZS), characterized by microcephaly and neurodevelopmental abnormalities [5].

The placental barrier, composed of specialized trophoblasts and immune-modulating decidual tissue, naturally protects the fetus [6]. However, ZIKV can bypass these defenses, leading to serious pregnancy complications. The “fetal-maternal interface,” which includes trophoblast layers (cytotrophoblasts, syncytiotrophoblasts, and extravillous trophoblasts), maternal decidual cells, and immune networks, facilitates nutrient exchange and tolerance of the semi-allogeneic fetus [7]. Disruption of this interface by ZIKV has been linked to placental dysfunction, fetal growth restriction, and increased miscarriage risk [8]. This review aims to address the knowledge gap by focusing on how ZIKV affects the fetal-maternal interface and outlining future strategies to understand ZIKV's long-term effects on fetal health and potential interventions.

## The fetal-maternal interface

The placenta is unique to pregnancy, ensuring fetal oxygenation and nutrition while balancing maternal immune tolerance [9]. On the fetal side, the placenta is composed of chorionic villi containing trophoblasts, which are essential for nutrient exchange and immune modulation [10]. These trophoblasts include three main lineages: cytotrophoblasts (CTBs), syncytiotrophoblasts (STBs), and extravillous trophoblasts (EVTs), which work together to sustain pregnancy [11]. CTBs are mononuclear progenitor cells that proliferate and differentiate into either STBs or EVTs. STBs formed from the fusion of CTBs, create the multinucleated outer layer of placental villi, directly interfacing with maternal blood to facilitate material exchange and act as a barrier against pathogens [12]. EVTs, derived from the epithelial–mesenchymal transition of CTBs, invade the maternal decidua and remodel spiral arteries to ensure adequate blood perfusion during pregnancy [13]. Hofbauer cells, which are fetal-origin macrophages within chorionic villi, regulate the response to infections, modulate the local immune environment, and support trophoblast function, thereby maintaining placental integrity [14]. On the maternal side, the decidua consists of maternal stromal cells, immune cells, and blood vessels, which collectively support implantation and placental development [15].

Non-trophoblast immune populations play pivotal roles in modulating ZIKV infection at the maternal-fetal interface. Decidual natural killer (NK) cells, which are the most abundant leukocyte in the decidua, can directly kill virus-infected trophoblasts to limit ZIKV spread; however, ZIKV can hinder this response by upregulating MHC class I [16,17]. Regulatory T cells (Tregs) help control excessive placental inflammation and limit bystander tissue damage, yet ZIKV-driven alterations in their function may shift the immune balance toward pathological states [18]. Meanwhile, dendritic cells (DCs) orchestrate adaptive immune responses by recruiting T lymphocytes and releasing interferons, but ZIKV can subvert DCs by suppressing their maturation and dampening interferon signalling [19,20]. Thus, beyond Hofbauer cells, these immune cell subsets collectively shape the placental immune environment. Elucidating their nuanced cross-talk and ZIKV’s evasion strategies will be crucial for designing interventions that protect against vertical transmission.

During a healthy pregnancy, the immune system must maintain a delicate balance: it needs to be sufficiently quiescent to prevent destructive inflammation but also ready to protect maternal tissues and fight off pathogens. However, ZIKV can disrupt this balance both at the maternal-fetal interface and throughout the body, allowing maternal and fetal tissues to coexist without triggering a significant immune response. Understanding the microbiological characteristics of ZIKV reveals why it can successfully breach the fetal-maternal barrier.

## ZIKV: an overview

### Virology

In 2023, the Zika virus, scientifically named *Orthoflavivirus zikaense*, was reclassified under the Ntaya virus group within the *Orthoflavivirus* genus of the *Flaviviridae* family [21]. This genus also includes other notable viruses such as Dengue (DENV), Japanese encephalitis (JEV), and yellow fever (YFV).

ZIKV has a positive-sense single-stranded RNA (+ssRNA) genome, classified under Group IV in the Baltimore classification system. The ZIKV virion features a ill-defined isometric capsid (C) containing its genome, which is approximately 10,700 nucleotides long [22]. This capsid is encased in a lipid membrane associated with envelope (E) and membrane (M) proteins. During maturation, larger immature virions, about 60 nm in diameter and characterized by prM-E (precursor of membrane-envelope protein trimers) protrusions, shrink and smoothen to a diameter of 50 nm as the prM protein is cleaved and the E protein homodimer reorients [22].

The ZIKV genome encodes a single open reading frame (ORF) that translates into a polyprotein. This polyprotein is subsequently cleaved into three structural proteins (C, prM, and E) and seven non-structural proteins (NS1, NS2A, NS2B, NS3, NS4A, NS4B, and NS5) in two major segments [22,23]. The genome is flanked by untranslated regions (UTRs) at both the 5’ and 3’ ends. The 5’ UTR is crucial for RNA-dependent RNA polymerase (RdRp) binding and activity, whereas the NS5-mediated type I (m7GpppAmp) methylation cap protects the RNA from nucleases and aids in immune evasion [24]. The 3’ UTR lacks a poly-A tail but folds into various structures, such as stem-loops and dumbbells, which protect it from nucleases and may influence pathogenesis [25,26].

The infection cycle of the ZIKV involves several stages, similar to other flaviviruses: (1) Attachment: The virus attaches to the host cell's surface through interactions between the viral envelope protein (E) and the host cell's adhesion factors and receptors. (2) Penetration: The virus enters the host cell primarily via clathrin-mediated endocytosis. (3) Uncoating: The viral genome is released from the virion into the host cell's cytoplasm in a pH-dependent process. (4) Translation: The viral genome is translated into a polyprotein, which is then cleaved into various viral proteins. (5) Replication: The viral RNA is replicated using the NS5 RNA-dependent RNA polymerase (RdRp) activity. (6) Assembly: New viral particles are assembled in the host cell's cytoplasm. (7) Release: The host cell releases the viral particles through exocytosis [27–29].

### Key mechanisms of ZIKV infection

ZIKV, like other flaviviruses, utilizes a complex series of interactions to enter host cells. The process begins with the attachment of the viral envelope protein (E) to specific host cell adhesion factors and receptors. AXL receptor tyrosine kinase (AXL) is a significant entry receptor for ZIKV [27,30], widely expressed in various cell types, including neural progenitor cells (NPCs), neuronal cells, fetal endothelial cells, and testicular cells, which aligns with ZIKV's neurotropism and sexual transmission [31,32]. TYRO3 protein tyrosine kinase (TYRO3), another member of the TAM family (TYRO3, AXL, and MERTK), and the CD209 molecule (DC-SIGN) are implicated in facilitating ZIKV entry [27]. Furthermore, T-cell immunoglobulin and mucin domain (TIM) family proteins, specifically hepatitis A virus cellular receptor 1 (HAVCR1) and T cell immunoglobulin and mucin domain containing 4 (TIMD4), also participate in the infection process by binding to phosphatidylserine (PS) and phosphatidylethanolamine (PE) on the viral envelope [28,33]. Beyond receptor usage, ZIKV’s capacity to breach the maternal-fetal barrier is mediated by structural adaptations that enhance placental tropism. These include its envelope (E) protein glycosylation site (Asn154), which facilitates binding to AXL and DC-SIGN receptors on trophoblasts; delayed prM cleavage, enabling stealthy transit across placental macrophages; and NS1-mediated suppression of trophoblast IFN-λ responses [28]. Additionally, a nuclear localization signal in the capsid protein promotes viral persistence in placental stromal cells. These functional sites collectively underscore ZIKV’s evolutionary optimization for vertical transmission and present targets for therapeutic intervention aimed at disrupting placental infection. Despite substantial research identifying these receptors as critical for ZIKV entry, conflicting evidence from AXL-knockout [34] and TAM-knockout mice models suggests the involvement of alternative pathways or co-receptors in viral infection in vivo, where AXL and TAM deficient mice show similar pathology to their littermates and wildtype control. These discrepancies highlight the virus’s complex, multifactorial invasion mechanism, which likely extends beyond AXL-dependent processes. Further investigation is required to elucidate these alternative entry routes and to advance the development of targeted therapeutic interventions aimed at receptor-mediated ZIKV pathogenesis. Given the recent surge in research interest in ZIKV biology, additional receptors likely facilitating ZIKV infection remain to be discovered. Future studies may benefit from examining established receptors and attachment factors in other well-studied flaviviruses, particularly members of the integrin family, laminin receptors, and heat shock protein family A [35,36].

#### Influence of co-infections and prior flavivirus exposure

Prior infections with flaviviruses, such as DENV and West Nile virus (WNV), have been associated with more severe infection outcomes due to the development of cross-reacting antibodies. This phenomenon has been suggested to contribute to ZIKV pathogenesis through mechanisms such as antibody-dependent enhancement (ADE), where cross-reactive antibodies promote viral entry through the Fcγ receptors and also play a part in immune evasion and modulation of immune responses. This is initially supported by observations of increased incidence of neurological malformations in neonates in regions where DENV is endemic [37–39]. As ZIKV establishes itself in areas where other arthropod-borne flaviviruses are endemic, understanding these potential mechanisms becomes essential. Recent studies have shown that cross-reactive DENV antibodies may increase the risk of maternal-fetal transmission by promoting the binding and entry of immune complexes formed between DENV antibodies and ZIKV virions, while also reducing the cell’s antiviral response mediated by interferons and cytokines [40–42]. In vitro experiments and mouse models using convalescent plasma from DENV and WNV patients have demonstrated significant enhancement of ZIKV viral entry mediated by flaviviral IgG, leading to higher rates of vertical transmission and adverse infection outcomes in mice [43–45]. However, further studies are needed to fully elucidate the underlying mechanisms, as many of these findings were derived from cell line and animal model studies, which have structural differences in placental composition compared to humans [46].

### Transmission routes

ZIKV is primarily transmitted through the bite of an infected Aedes mosquito, typically *Aedes aegypti*. However, recent mutations have enabled ZIKV to be carried by other mosquito species, such as *Culex quinquefasciatus*, which inhabit tropical and subtropical regions and can potentially spread the virus further [47,48]. In sylvatic habitats, mosquitoes circulate ZIKV among non-human primates, including rhesus monkeys, creating an enzootic cycle and acting as a reservoir gene pool [49,50]. ZIKV is also transmitted to humans through the bites of infected mosquitoes, forming an epidemic cycle when the virus spreads among susceptible individuals [49,50]. Additionally, ZIKV can be transmitted through sexual intercourse, blood transfusions, vertical transmission from mother to fetus during pregnancy, and contact with infectious urine or sewage, further sustaining the epidemic cycle [23,51,52].

### ZIKV tropism and replication dynamics at the fetal-maternal interface

ZIKV exhibits distinct cellular tropism at the fetal-maternal interface, targeting progenitor trophoblasts, decidual stromal cells, fetal endothelial cells, and Hofbauer cells. Once internalized, ZIKV replicates within the endoplasmic reticulum (ER), using non-structural proteins (NS2A/NS4A, NS3/NS5) to form replication complexes [24,27]. Undifferentiated CTBs exhibit high replication rates, partly due to weak induction of interferon-stimulated genes (ISGs). Importantly, these observations have been cooroborated beyond cell lines: trophoblast stem cells and 3D trophoblast organoids derived from first-trimester placental tissue show identical susceptibility patterns and interferon responses [53]. Additionally, the NS5 protein inhibits type I interferon responses by degrading signal transducer and activator of transcription 2 (STAT2) [54]. The high susceptibility is also attributed to the high expression of viral entry receptors like AXL and TIM-1 [27,55]. Functional experiments using CRISPR-Cas9 in trophoblast organoids have shown that knocking out AXL reduces viral entry, while overexpressing AXL in more resistant cells increases their susceptibility to ZIKV [53,55].

Decidual stromal cells express AXL, PROS1, and GAS6, which bind to TAM receptors [56,57]. Once infected, these cells can transmit the virus to adjacent cytotrophoblasts through receptor-mediated endocytosis [58,59]. ZIKV also targets fetal endothelial cells and Hofbauer cells, indicating the virus’s ability to exploit various placental and fetal cells for vertical transmission [60]. Hofbauer cells can sustain a persistent infection without significant inflammation [61], potentially allowing the virus to remain near the fetal circulation for extended periods.

STBs, forming a continuous layer in direct contact with maternal blood, exhibit higher resistance to infection due to robust expression of type III interferons (IFNλ1) and a lower surface density of AXL [53,62]. In contrast, other placental cells, such as cytotrophoblasts (CTBs) and Hofbauer cells, are more susceptible to ZIKV infection due to their higher expression of viral entry receptors and weaker antiviral defenses. This heterogeneity in susceptibility among different placental cell types explains how infections can originate in focal areas of the placenta and then spread to broader maternal or fetal compartments. In addition, several viruses in the Flaviviridae family are known to exploit integrins or heat-shock proteins for cell entry. Further research is needed to determine whether the ZIKV employs a similar mechanism.

## Impact on pregnancy outcomes and fetal health

Despite approximately 80% of ZIKV infections in humans are asymptomatic, the ZIKV epidemic has had significant clinical implications, particularly regarding congenital Zika syndrome (CZS), neurological development, and pregnancy outcomes. Symptomatic patients typically experience acute infections characterized by non-specific “flu-like” symptoms, including mild fever, joint and muscle pain, headache, fatigue, abdominal pain, swelling, lymph node enlargement, retro-orbital pain, conjunctivitis, and rash. Some patients also suffer from moderate thrombocytopenia, with certain cases progressing to significant subcutaneous bleeding [63,64]. Neurological complications, such as self-limiting meningoencephalitis and Guillain-Barré syndrome, have also been observed, as is common with other arboviruses.

ZIKV has been increasingly linked to neurological complications and fetal central nervous system malformations. Brazilian health authorities noted a dramatic rise in severe neonatal microcephaly cases, which often resulted in developmental delays or, in severe instances, cerebral palsy [65]. This connection was confirmed when studies detected ZIKV sequences in the brain tissues of microcephalic fetuses from mothers who had reported symptoms [66]. Adverse fetal outcomes, including fetal deaths, growth restrictions, microcephaly, ventricular calcifications, other CNS lesions, and abnormal amniotic fluid volume or artery flow, have been reported in up to 30% of infected pregnant women. These clinical observations are supported by direct evidence from human studies, such as ZIKV sequences were detected in the brain tissues of microcephalic fetuses from infected mothers using RT–PCR and electron microscopy, confirming vertical transimission and its teratogenic effects [66]. Similar findings were retrospectively reported during the 2013–2014 French Polynesia outbreak [67].

Systemic maternal immune activation (MIA) triggered by ZIKV infection may further exacerbate adverse fetal neurodevelopmental outcomes. Elevated maternal pro-inflammatory cytokines, such as IL-6, IL-1β and TNF-α, have been shown to cross the placenta, potentially altering fetal brain development and contributing to conditions like microcephaly and neurodevelopmental delays [68,69]. These cytokines can disrupt critical processes such as synaptic pruning, neuronal migration, and myelination, compounding the teratogenic effects of direct ZIKV infection in the fetal brain [70]. Moreover, animal models have shown that maternal inflammation during gestation can lead to long-term behavioural and cognitive deficits in offspring, linking MIA interacts with direct viral effects to influence ZIKV-induced neurological outcomes [71].

Beyond overt microcephaly, some infants exposed to ZIKV in utero exhibit subtle developmental delays, sensory impairments, or immune dysregulation that may only manifest later [3]. While the recent ZIKV epidemics in the Americas were strongly associated with a surge in congenital microcephaly, the virus's neurotropic nature and its targeting of neural progenitor cells during fetal development, little is known about the spectrum of effects resulting from in utero exposure to ZIKV in fetuses born with a normal head circumference [66,72–74]. Initial studies on this issue present conflicting findings. Some suggest that normocephalic ZIKV-exposed infants generally exhibit no significant differences in neurodevelopmental outcomes, while others report subtle deficits in language, cognitive, and motor skills, as well as potential vulnerabilities in the visual system, which warrant continued monitoring and early intervention [75–77]. Extensive research in this area is still lacking, as accurately diagnosing prior ZIKV infections remains challenging due to the transient viremia stage and short-lived immunoglobulin M (IgM) responses. These limitations have hindered the development of comprehensive studies with well-designed control groups. Additional challenges arise from the lack of culturally and linguistically appropriate neurodevelopmental assessments for infants in South America [78]. Advances from non-human primate (NHP) infection models have revealed distinctive injury patterns and attenuated neurogenic outputs when infection occurs during pregnancy, even in the absence of apparent microcephaly, underscoring the need for more extensive research in exposed populations [79]. Rodent and primate models suggest that even low-grade infection or subclinical maternal illness could impact fetal organogenesis, particularly the central nervous system [7]. Therefore, studying older children who were prenatally exposed to ZIKV is crucial to understand the full extent of its clinical impact.

In addition to early-gestational infection, studies have consistently indicated that ZIKV exposure in mid- and late-gestation can produce a broad spectrum of fetal injuries. For instance, mid-gestation infection can trigger neuropathological changes including gliosis and white matter injury, as indicated in non-human primate and ovine models [80–82]. Notably, some examinations reveal sustained viral replication in placental and fetal tissues during mid-pregnancy, leading to neural precursor cell depletion, neuroinflammation, and disruption of radial glial fibre architecture [83]. Furthermore, late-gestational exposures, documented can still result in neurodevelopmental anomalies in various animal models and accompanied by placental insufficiency and inflammatory pathologies that diminish fetal oxygenation [60,84]. While microcephaly and severe malformations are typically linked to infections at or near the first trimester, these later stages can also see subtler cortical and subcortical disruptions, as well as postnatal sequelae such as impaired myelination and potential neurobehavioral deficits. Consequently, expanding our discussion to encompass mid- and late-gestational findings reinforces that adverse outcomes are not confined exclusively to first-trimester infection but can extend throughout pregnancy. Moreover, ZIKV infection can impair spiral artery remodelling by infecting EVTs, which reduces blood flow to the placenta and limits nutrient and oxygen exchange. This topic will be explored in the following sections.

## ZIKV-induced cellular responses in trophoblast

Early gestational ZIKV infection may increase the risk of miscarriage by targeting the trophectoderm at the blastocyst stage. Studies using murine models show that pre-implantation infection leads to transcriptional dysregulation in blastocysts and reduced pregnancy rates, suggesting that viral replication at these early developmental stages can cause embryonic demise [8]. Emerging clinical and mechanistic evidence also indicates that ZIKV disrupts placental homeostasis, leading to adverse pregnancy outcomes. This section explores key mechanisms underlying cellular responses in trophoblasts after ZIKV infection (Table 1).

**Table 1.** Key studies investigating ZIKV infection mechanisms and outcomes in placental and decidual models.

Author/Date	Title / Major Findings	Viral Strains	Experimental Methods	Experimental Model
Bayer et al, [62]	Type III Interferons Produced by Human Placental Trophoblasts Confer Protection against Zika Virus Infection [PMID: 27066743] Trophoblasts constitutively produce interferon lambda 1 (IFNλ1), a type III interferon, which protects non-placental cells by inducing interferon-stimulated genes (ISGs) to inhibit ZIKV replication. Blocking IFNλ1 signalling partially restores ZIKV infection susceptibility.	ZIKV MR766 (Ugandan origin); ZIKV FSS13 025 (Cambodian origin);	ZIKV infection in vitro (MR766 & FSS13025) at 1–3 focus-forming units/cell IFNλ1 neutralization ISG induction measured by qRT-PCR and antiviral reporter assays	**Cel lines**: BeWo, JEG-3, JAR, HTR8/SVneo human trophoblast cell lines **Primary (ex vivo**): Term primary human trophoblasts isolated from healthy placentas
Quicke et al, [61]	Zika Virus Infects Human Placental Macrophages [PMID: 27247001] Hofbauer cells are more permissive to ZIKV infection than cytotrophoblasts. ZIKV infection induces antiviral gene expression, but responses vary between donors. Discordance between viral RNA levels and infectious virus production suggests donor-specific regulation.	ZIKV PRVABC59	ZIKV PRVABC59 infection at MOI 1 Antiviral gene profiling by qRT-PCR Viral replication quantified by focus-forming assay and qRT-PCR	**Primary (ex vivo)**: Human placental macrophages (Hofbauer cells) and cytotrophoblasts isolated from chorionic villi of full-term placentae
Pagani et al, [113]	Human Endometrial Stromal Cells Are Highly Permissive To Productive Infection by Zika Virus [PMID: 28281680] Decidualized endometrial stromal cells exhibit 100-fold increased ZIKV replication, linked to AXL receptor upregulation. ZIKV triggers delayed IFN-β and ISG responses in decidualized cells. ZIKV utilizes vimentin filaments and endoplasmic reticulum for replication.	MR766 (African strain); INMI-1 (Brazilian 2016 isolate); PRVABC59 (Puerto Rico 2015); Thailand 2013	Hormonal decidualization: progesterone + cAMP AXL receptor expression by cytofluorimetric analysis The kinetics of virus replication in T-HESC and dT-HESC cells were measured by plaque forming assay	**Cell lines**: Primary human endometrial stromal cells (HESC); telomerase-immortalized (T-HESC) human endometrial stromal cell line; in vitro decidualization of the T-HESC cell (dT-HESC)
Puerta-Gurado et al, [95]	Zika Virus Nonstructural Protein 1 Disrupts Glycosaminoglycans and Causes Permeability in Developing Human Placentas [PMID: 31250000] ZIKV NS1 reduces heparan sulfate, hyaluronic acid (HA), and sialic acid expression in trophoblasts, causing shedding. NS1 increases placental permeability in early gestation (7-9 weeks) and upregulates hyaluronidase (HYAL). NS1 modulates HA receptor CD44 and LYVE-1 expression, disrupting placental barrier integrity.	ZIKV Nica 2–16	Treatment with purified ZIKV NS1 protein (5 µg/mL) Glycosaminoglycan staining (immunofluorescence for HS, HA, sialic acid) Dextran-fluorescence permeability assay	**Cell line**: JAR, JEG-3 human trophoblast cell lines **Primary (ex vivo)**: First trimester placental explants (7-14 weeks gestational age)
Tan et al, [8]	Pre- and Peri-implantation Zika Virus Infection Impairs Fetal Development by Targeting Trophectoderm Cells [PMID: 31519912] ZIKV infects trophectoderm cells (TECs) during pre-/peri-implantation causes caspase-3-mediated cell death. High ZIKV doses exposure kills TECs; low doses exposure allows viral propagation to fetal neurons. Pre-implantation infection increases miscarriage risk; post-implantation infection damages neural progenitors.	ZIKV MR766; ZIKV FSS13025	Mouse and human pre-implantation embryos were infected with the MR766 strain ex vivo In vivo infection of pregnant interferon-alpha/beta receptor (IFNAR) antibody-treated C57BL/6 mice (subcutaneous at E2.5) measuring embryo loss at E6.5	**Cell lines**: Human embryonic stem cells and human neural progenitor cells (hNPC) **Animal (in vivo)**: Pregnant C57BL/6 mice (with IFNAR antibody treatment) infected at a pre-implantation stage (E2.5); pregnant Ifnar-/- mice infected at pre-implantation (E2.5, E3.5) or peri-implantation (E4.5) stages
Rabelo et al, [97]	Zika Induces Human Placental Damage and Inflammation [PMID:32983175] ZIKV causes placental inflammation, delayed villi maturation, and mitochondrial damage in cytotrophoblasts. Increased macrophages, CD8+ T cells, and pro-inflammatory cytokines (TNF-α, IFN-γ) in placenta Extracellular matrix degradation via matrix metalloproteinase (MMP)-2/9 leads to vascular dysfunction.	ZIKV MR 766	ZIKV strain MR 766 for the plaque-reduction neutralization test (PRNT90%) Cytokine/chemokine levels were profiled using immunohistochemistry Histopathology and immunohistochemistry for immune cell infiltration	**Primary (ex vivo)**: Term human placental tissue samples **Cell line**: Vero E6 (African green monkey kidney)
Muthuraj et al, [87]	Zika Virus Infection Induces Endoplasmic Reticulum Stress and Apoptosis in Placental Trophoblasts [PMID: 33500388] ZIKV activates endoplasmic reticulum (ER) stress pathways (PERK, IRE1α) and induces caspase-dependent apoptosis. JNK activation promotes viral replication and apoptosis; ER stress inhibitors (e.g. salubrinal) reduce ZIKV-induced cell death. ZIKV evades innate immunity by modulating RIG-I-like receptor signalling.	MR766; recombinant MR strain (r-MRV); PRVABC59	Infection with MR766, r-MRV & PRVABC59 at MOI 0.1-1 ER stress inhibition with salubrinal (20 µM) JNK activation by phospho-JNK byWestern blot analysis Apoptosis by caspase-3/7 assay	**Cell line**: HTR-8/SVneo, JEG-3, JAR trophoblast cell lines
Zhao et al, [68]	Zika Virus Causes Placental Pyroptosis and Associated Adverse Fetal Outcomes by Activating GSDME [PMID:35972780] ZIKV induces pyroptosis in JEG-3 cells via gasdermin E (GSDME) cleavage by caspase-3. Mechanism: RIG-I detects viral RNA → TNF-α → caspase-8/3 → GSDME activation. Blocking TNF-α with R-7050 reduces placental damage in mice.	ZIKV strain H/PF/2013	CRISPR/Cas9 knockout of retinoic acid- inducible gene I (RIG-I), Melanoma Differentiation-associated protein 5 (MDA5) & Mitochondrial Antiviral Signaling protein (MAVS) in JEG-3 Pharmacological TNF-α blockade (R-7050) Mouse placentas harvested for histology and fetal outcome was measured Pyroptosis assays (LDH release, GSDME cleavage by Western blot analysis)	**Cell lines**: JEG-3 trophoblast with wild-type (WT) and RIG-I /MDA5/MAVS KO variants **Animal (in vivo)**: Pregnant immunocompetent C57BL/6 mice infected at mid-gestation
Cervantes et al, [86]	Zika Virus Infection Induces Expression of NRF2 and Antioxidant Systems in Trophoblast Cells [PMID: 37326824] ZIKV upregulates nuclear factor erythroid 2-related factor 2 (NRF2), a stress-response transcription factor. Antioxidant enzyme activation counteracts oxidative stress in trophoblasts. NRF2 modulates host metabolism and innate immunity to mitigate ZIKV-induced placental damage.	Puerto Rico strain PRVABC59	NRF2 expression by Western blot analysis Antioxidant enzyme (SOD and CAT) expression levels measured by Western blot and cellular distribution examined by immunofluorescence. Oxidative stress markers (MDA and protein carbonylation) measured to indirectly assess ROS presence	**Cell line**: HTR-8/SVneo and JEG-3 trophoblast cell lines
Wu et al, [53]	Zika Virus Targets Human Trophoblast Stem Cells and Prevents Syncytialization in Placental Trophoblast Organoids [PMID: 37684223] Human trophoblast stem cells (hTSCs) are highly susceptible to ZIKV due to AXL/TIM-1 expression and low interferon (IFN) responses. ZIKV disrupts syncytialization in trophoblast organoids, mimicking preeclampsia. Single-cell RNA-seq shows reduced hTSC stemness and cytotrophoblast proliferation.	ZIKV FSS 13025 strain; ZIKV GZ01 strain	AXL/TIM-1 expression by qRT-PCR IFN/ISG profiling by bulk RNA-seq Single-cell RNA-seq of organoids	**In vitro**: Human trophoblast stem cells (hTSCs) **Ex vivo organoid**: Trophoblast organoids generated hTSCs
Kafer et al, [92]	Targeting first trimester trophoblast cell metabolism modulates its susceptibility to Zika virus infection [PMID: 36790938] ZIKV infection alters trophoblast metabolism (increased glucose use, lipid droplet accumulation). Vasoactive intestinal peptide (VIP) inhibits ZIKV replication and restores cell migration. Blocking VIP increases AXL receptor expression and viral particle production.	ZIKV Argentinian isolate INEVH116141	Metabolic flux: measuring glucose uptake using the fluorescent probe 2-NBDG, and assessing lipid droplet abundance using the fluorescent probe BODIPY 493/503 VIP treatment (1–50 nM) and VIP receptor blockade using VIP receptor antagonist (VIPant, 50–100 nM) AXL surface expreesion by RT-qPCR	**Cell lines**: Swan71 and HTR-8/SVneo trophoblast cell lines

### Oxidative and Endoplasmic Reticulum (ER) stress

ZIKV disrupts trophoblast function by inducing reactive oxygen species (ROS) and activating nuclear factor erythroid 2-related factor 2 (NRF2)-driven antioxidant pathways. This oxidative stress response is evidenced by the increased expression of antioxidant enzymes, such as superoxide dismutase 1 (SOD-1) and catalase (CAT), which redistribute to sites of viral presence within infected cells to counteract oxidative damage [85,86]. Concurrently, viral replication within ER membranes can trigger ER stress, marked by the phosphorylation of the eukaryotic initiation factor 2 alpha subunit (eIF2α) and activation of the Isothiol-Requiring enzyme 1 α (IRE1α)—X-box binding protein 1 (XBP1) axis [87]. Persistent or intense ER stress leads to the upregulation of the transcription factor C/EBP homologous protein (CHOP), which triggers the apoptotic cascade, resulting in the thinning or fragmentation of the syncytium [88,89]. This tissue injury compromises the placental barrier, facilitating viral invasion and transmission to fetal tissues.

### Pyroptosis

In addition to classical apoptosis, ZIKV induces an alternative inflammatory cell death pathway called pyroptosis, mediated by the caspase-8/-3 cascade and gasdermin E [68,90,91]. Activation of the retinoic acid-inducible gene I (RIG-I) by viral RNA triggers cascade events, including inflammasome assembly, caspase activation, and gasdermin E cleavage, leading to plasma membrane pore formation. Pyroptotic cells release pro-inflammatory cytokines and damage-associated molecular patterns (DAMPs), exacerbating local inflammation. Crucially, this mechanism has been validated in vivo: Zhao et al. demonstrated in a pregnant mouse model that ZIKV infection activates GSDME-mediated pyrotopsis in trophoblasts, and that pharmacological blockade of TNF-α mitigates fetal demise [68]. Moreover, examination of human placental tissue from adverse outcomes shows markers of pyroptotic death, including cleaved gasdermin E and elevated TNF-α, alighing with these pre-clinical findings. This process can create micro pockets of compromised barrier integrity within the placenta, facilitating vertical transmission.

### Metabolic reprogramming

Flaviviruses typically exploit host lipid droplets to sustain viral replication [92]. In first-trimester trophoblasts, enhanced glycolysis and lactate production create a Warburg-like effect that supports viral biosynthesis. Additionally, changes in mitochondrial membrane potential and reduced uptake of long-chain polyunsaturated fatty acids compromise overall cellular function [92]. ZIKV also reprogrammes the lipidome and altering diacylglycerol and triacylglycerol metabolism, which impairs the placenta's metabolic capacity to support fetal growth [93]. The infection exacerbates mitochondrial dysfunction and reduces ATP production, diminishing the energy required for active nutrient transport [94]. These combined effects result in fetal growth restriction (FGR), even in the absence of overt fetal neuropathology, and lead to long-term developmental deficits due to persistent placental hypoperfusion [69].

### Disruption of placental integrity

ZIKV encodes a secreted NS1 protein that degrades glycosaminoglycans and alters tight-junction proteins such as ZO-1 and E-cadherin, increasing paracellular permeability and compromising barrier function [95,96]. These effects were first observed in JAR and JEG-3 cells, but have since been confirmed in ealy-gestation human placental explants (7–9 weeks) exposed to recombinant NS1, which exhibit reduced heparan sulfate, increased hyaluronidase expression, and barrier leakage ex vivo [96]. Concurrently, matrix metalloproteinases (MMP-2, MMP-9) released during infection degrade collagen in chorionic villi, further reducing vascular integrity [97,98]. The combined effects of these morphological disruptions and vascular compromise substantially diminish placental efficiency. Evidence from trophoblast organoids shows that ZIKV also impairs STB differentiation and reduces the expression of fusogenic genes like syncytin-1 and syncytin-2, along with their transcriptional regulators GCM1 and OVOL1 [53,99,100]. Incomplete syncytialization not only weakens the physical barrier but also disrupts the production of human chorionic gonadotropin (hCG) and placental lactogens, potentially leading to fetal growth restriction or pregnancy complications [99,101].

### Trophoblast-derived Type III interferons and immune modulation

Type III interferons (IFN-λ) are increasingly recognized for their specialized role in placental and epithelial defenses. Unlike type I (IFN-α/β) and type II (IFN-γ) interferons, which broadly activate a systemic antiviral programme affecting many cell types, type III IFNs generally focus on mucosal and barrier tissues, such as those at the maternal-fetal interface. STBs constitutively produce type III interferons (IFNs), particularly IFNλ1, without requiring prior viral recognition signals [62]. IFNλ1, through autocrine and paracrine actions, activates interferon-stimulated genes (ISGs) to create a strong antiviral environment that protects both trophoblasts and neighbouring cells from ZIKV. This defense is crucial for maintaining placental immunity and protecting the maternal–fetal interface. However, ZIKV employs several evasion strategies to counteract these defenses. The virus's NS5 protein degrades STAT2, undermining type I IFN signalling and enhancing viral replication [54]. In addition, NS2A and NS4A proteins inhibit IFN-β production by disrupting promoter activity [102]. ZIKV also modulates immune-related pathways and microRNA expression, impairing RIG-I-like receptor (RLR)-mediated antiviral defenses [103]. Through these mechanisms, ZIKV establishes a reservoir within the placenta, facilitating vertical transmission.

### Therapeutic strategies

Multiple studies have now demonstrated pre-clinical evidence that certain therapeutics can mitigate ZIKV–related placental infection and associated fetal outcomes. In a mouse model of in utero infection [104], maternal treatment with an IL-1 receptor antagonist (anakinra) not only preserves placental function (e.g. trophoblast invasion, vascular remodelling) but also increases fetal viability, reduces neurobehavioral deficits in offspring, and lowers neuroinflammation. This suggests that postpartum neuroinflammatory processes—and their unfavourable developmental consequences—can be dampened by targeting IL-1 signalling during pregnancy. Another therapeutic approach arises from autophagy inhibition. Data from pregnant mice and pregnant women report that hydroxychloroquine (an inhibitor of autophagy) reduced both placental and fetal ZIKV infection, strengthening the rationale for modulating cellular pathways as a means to block vertical transmission [105]. Beyond these immunologic or cellular interventions, small-molecule inhibitors have also been proposed. For instance, nucleoside/nucleotide analogs and nonnucleoside inhibitors [106] have shown anti-ZIKV activity in vitro and—if advanced to in vivo studies—could further expand the repertoire of compounds that may provide placental or fetal benefit. Collectively, these findings indicate that multiple classes of therapeutics (e.g. immunomodulators, autophagy blockers, and antiviral small molecules) display promising efficacy in pre-clinical models to reduce placental ZIKV infection and safeguard fetal outcomes.

Recent studies suggest that antibody-based therapies represent a promising avenue for mitigating ZIKV disease, including maternal-fetal complications. Specifically, neutralizing monoclonal antibodies have been shown to reduce viral burden and protect fetuses in pregnant mouse models [107]. For example, human-derived monoclonal antibody ZIKV-117 not only broadly neutralized multiple strains of ZIKV, but also conferred significant protection by suppressing placental infection, fetal disease, and mortality when administered either prophylactically or shortly after infection. These effects were observed in a pre-clinical setting where pregnant mice had treatments that limited their interferon response, a known barrier to ZIKV pathogenesis studies in standard wild-type mice. If these data are recapitulated in non-human primates and then translated safely to pregnant humans, passive immunoprophylaxis with such antibodies might help protect at-risk pregnancies from severe ZIKV outcomes.

## Discussion

Despite advances in understanding ZIKV’s interaction with the fetal-maternal interface, critical gaps persist. Outstanding questions include how ZIKV evades immune surveillance in placental niches, the role of maternal-fetal genetic/epigenetic variability in susceptibility, and trimester-specific risks for subclinical neurodevelopmental sequelae. Additionally, the interplay between ZIKV and preexisting flaviviral immunity remains poorly defined, particularly in endemic regions. Key challenges such as the limited translatability of in vitro models to human pathophysiology, ethical constraints in clinical sampling, and the absence of pregnancy-safe antivirals hinder the research progress. Addressing these gaps requires interdisciplinary efforts integrating advanced placental models, longitudinal clinical cohorts, and targeted therapeutic screening to mitigate the enduring burden of congenital Zika syndrome.

Advancements in modelling techniques are crucial. Beyond traditional two-dimensional trophoblast cultures, three-dimensional organoid systems can accurately replicate early placental architecture. This allows for direct observation of ZIKV spread from anchoring villi to syncytial layers [53]. To meet future needs, multi-lineage “assembloid” systems and microfluidic “organ-on-a-chip” technology simulating maternal blood flow will be essential [108]. Integrating these organoids with microfluidic platforms will enable dynamic modelling of ZIKV-host interactions by replicating maternal-fetal blood flow [109]. Complementary in vivo imaging studies in non-human primates, which more closely resemble the human reproductive system than rodents, could further clarify how ZIKV transfers from maternal blood to fetal tissues.

Emerging therapeutic strategies targeting ZIKV-placental interactions show potential to disrupt viral pathogenesis while preserving fetal-maternal tolerance. For instance, vasoactive intestinal peptide (VIP) not only suppresses ZIKV replication by enhancing BST-2-mediated viral retention and downregulating AXL receptors but also safeguards trophoblast metabolic homeostasis [92,110–112]. Similarly, ER stress modulators like salubrinal protect against infection-induced apoptosis, and pyroptosis inhibitors may limit inflammatory placental damage [87]. Decidual stromal cell vulnerability to ZIKV could be countered by AXL-targeted therapies, preventing viral entry at the maternal-fetal interface [113]. However, these interventions must be rigorously evaluated for dual efficacy, i.e.: blocking vertical transmission without disrupting immune or metabolic crosstalk critical for healthy pregnancy.

Despite recent advancements, the cornerstone of ZIKV control remains effective vector management in endemic regions and vaccination strategies [114]. Although ZIKV vaccines are not yet widely implemented, several candidates have progressed in clinical trials. Integrated mosquito management, environmental sanitation, and community education are equally critical [115]. Vigilant clinical practices, such as RT–PCR testing of maternal blood and ultrasound surveillance, can also help identify ZIKV-related complications early, allowing for timely intervention.

**Figure 1.**
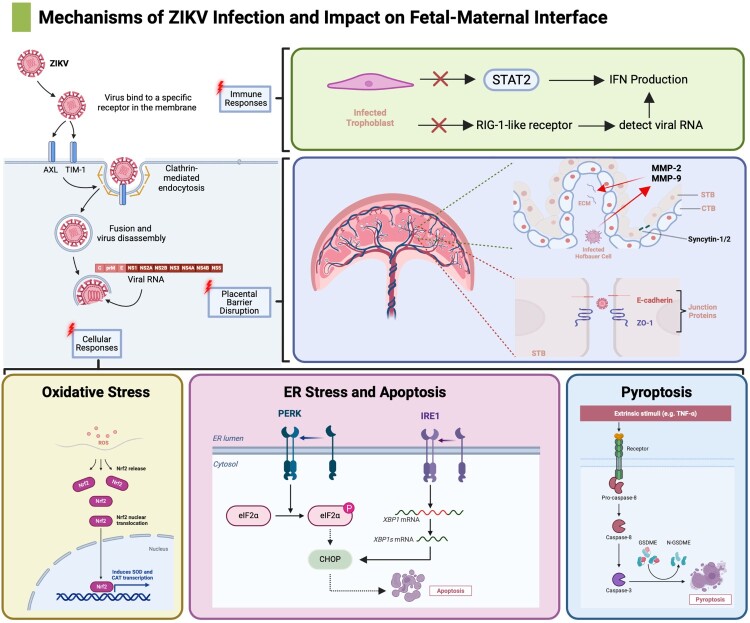
Mechanisms of Zika Virus (ZIKV) Infection and Impact on Fetal-Maternal Interface. In this schematic, four numbered steps indicate the key stages of ZIKV pathogenesis at the placental barrier: (1) Viral Entry: ZIKV enters trophoblasts via receptor-mediated endocytosis, primarily binding to AXL and TIM-1 receptors. (2) Immune Evasion: ZIKV impairs RIG-I-like receptor (RLR)-mediated antiviral defenses, and its non-structural protein NS5 degrades STAT2, suppressing type I interferon (IFN) responses. (3) Placental Barrier Disruption: Infection destabilizes syncytiotrophoblast (STB) and cytotrophoblast (CTB) integrity by downregulating junctional proteins (ZO-1, E-cadherin) and fusogenic factors (Syncytin-1/2). Matrix metalloproteinases (MMP-2/9) degrade extracellular matrix components, increasing paracellular permeability. (4) Cellular Stress Responses: ZIKV induces oxidative stress (NRF2 pathway), endoplasmic reticulum (ER) stress (PERK/eIF2α phosphorylation and IRE1/XBP1 splicing), apoptosis (CHOP activation), and pyroptosis (TNF-α-mediated caspase-3/-8 cleavage of gasdermin E). Collectively, these mechanisms disrupt placental function, enabling vertical transmission and contributing to fetal developmental abnormalities such as microcephaly and growth restriction.

In summary, ZIKV exhibits a remarkable ability to bypass placental defenses, causing immediate and long-term damage to fetal development (Figure 1). Understanding the mechanisms by which the virus infects trophoblasts and the multi-cellular niches at the fetal-maternal interface is crucial for developing effective preventative and therapeutic strategies. Collaboration across disciplines, including virology, immunology, obstetrics, pediatrics, and public health, is essential to enhance diagnostic tools, accelerate vaccine development, and create innovative treatments to reduce the global impact of ZIKV on maternal-fetal health.
